# Illusions of Visual Motion Elicited by Electrical Stimulation of Human MT Complex

**DOI:** 10.1371/journal.pone.0021798

**Published:** 2011-07-13

**Authors:** Andreas M. Rauschecker, Mohammad Dastjerdi, Kevin S. Weiner, Nathan Witthoft, Janice Chen, Aslihan Selimbeyoglu, Josef Parvizi

**Affiliations:** 1 Department of Neurology and Neurological Sciences, Stanford University, Stanford, California, United States of America; 2 Medical Scientist Training Program and Neurosciences Program, Stanford University, Stanford, California, United States of America; 3 Psychology Department, Stanford University, Stanford, California, United States of America; French National Centre for Scientific Research, France

## Abstract

Human cortical area MT^+^ (hMT^+^) is known to respond to visual motion stimuli, but its causal role in the conscious experience of motion remains largely unexplored. Studies in non-human primates demonstrate that altering activity in area MT can influence motion perception judgments, but animal studies are inherently limited in assessing subjective conscious experience. In the current study, we use functional magnetic resonance imaging (fMRI), intracranial electrocorticography (ECoG), and electrical brain stimulation (EBS) in three patients implanted with intracranial electrodes to address the role of area hMT^+^ in conscious visual motion perception. We show that in conscious human subjects, reproducible illusory motion can be elicited by electrical stimulation of hMT^+^. These visual motion percepts only occurred when the site of stimulation overlapped directly with the region of the brain that had increased fMRI and electrophysiological activity during moving compared to static visual stimuli in the same individual subjects. Electrical stimulation in neighboring regions failed to produce illusory motion. Our study provides evidence for the sufficient causal link between the hMT^+^ network and the human conscious experience of visual motion. It also suggests a clear spatial relationship between fMRI signal and ECoG activity in the human brain.

## Introduction

The posterior temporal region of the non-human primate brain (areas MT/MST), and its human homologue, known as area V5 [1] or human MT complex (hMT^+^) [2], [3] are responsive to visual motion [4]. Electrical stimulation of this region in non-human primates can influence motion direction discriminations, suggesting that its activity is critically linked to perceptual decisions [5], [6]. Although fundamental to our current understanding of motion perception, studies in non-human primates cannot ascertain conscious perceptual experiences during these direct alterations of neural activity.

To determine whether a brain region is causally linked to a perceptual experience, one must modulate its neural activity. Causal necessity can be established by inactivation (e.g. lesion) of the brain region and observing a perceptual deficit, whereas causal sufficiency is established by modulating its activity (e.g. by electrical stimulation) and observing a corresponding change in the perceptual experience. Non-invasive methods such as functional magnetic resonance imaging (fMRI) have provided evidence in the human brain of relationships between hMT^+^ responses and subjective visual motion perception (for review, see [7]). However, correlational techniques like fMRI and electroencephalography (EEG) cannot establish a causal relationship between hMT^+^ activity and conscious motion perception.

Non-human primate lesion studies first demonstrated the necessary role of MT in motion discrimination judgments [8], [9]. Subsequent reports addressed the necessity of human MT^+^ in the conscious experience of visual motion. For instance, visual motion blindness (akinetopsia) was reported in a few patients with extensive stroke in the posterior temporal region [10]–[12]. Deficits in motion processing have since been reported in healthy controls during transcranial magnetic stimulation (TMS) of posterior temporal cortex [13]–[16], in one patient with epilepsy during electrical stimulation of the anatomical area around hMT^+^, including superior, middle and inferior temporal and angular gyrus [17], and in a few patients with variable amounts of brain damage in the vicinity of the anatomical locus of hMT^+^ [18]–[24].

In contrast to these findings of disruption of motion perception, reports of positive percepts caused by functional alteration of hMT^+^ are missing [25]. Although some studies in humans have elicited “motion percepts” by electrical stimulation in various regions of the brain, the precise anatomical location of these stimulation sites and their spatial relationships to hMT^+^ remain uncertain. Penfield first reported illusory motion caused by electrical brain stimulation (EBS) of the posterior temporal region in some cases of intraoperative monitoring [26]. Plant and colleagues [22] reported a patient who saw a moving colorless “fog”, without moving objects, during seizure auras as well as during electrical stimulation of epileptic tissue. Lee and colleagues [27] reviewed the evidence of visual illusions caused by electrical stimulation of human visual cortex and suggested that the experience of “visual movement” can be elicited at many sites across cortex. We note, however, that the definition of visual movement was not specified. In a study of one patient implanted with intracranial electrodes, Matsumoto and colleagues [28] were the first to relate evoked potentials from magnetoencephalography (MEG) during a visual motion task with a patient’s reported illusions of objects moving in depth during electrical stimulation of the posterior superior temporal sulcus.

These previous findings of positive percepts must be interpreted with caution due to several caveats. The cortical tissue causing illusory percepts could have been diseased (epileptogenic), and the presence or absence of epileptic after-discharges (triggered by EBS) was not reported. In addition, the precise location of the stimulation was not adequately established by neuroimaging methods. Indeed, a more recent study failed to produce a visual motion percept by electrical stimulation at the border of fMRI-defined hMT^+^ in one patient [29], leaving open the question of whether electrical stimulation of hMT^+^ is sufficient to induce visual perceptions.

The question of the spatial relationship between effective sites of induction of visual illusions by EBS and the site of visual stimulus-induced activity recorded by fMRI and electrocorticography (ECoG) remains unexplored. Moreover, the relationship between fMRI and ECoG signals during motion perception has not been characterized but has the potential to provide a bridge between human fMRI measures and electrophysiological recordings in animals [30]. Combining three methods of neuroscientific inquiry (i.e. fMRI, ECoG, and EBS) in the same conscious human subjects allowed us to address the critical link between fMRI and electrophysiological correlates of motion perception and the role of hMT^+^ in the conscious perception of motion.

## Results

### Co-localization and pattern of responses to motion as measured by BOLD fMRI and ECoG

**Figure 1:**
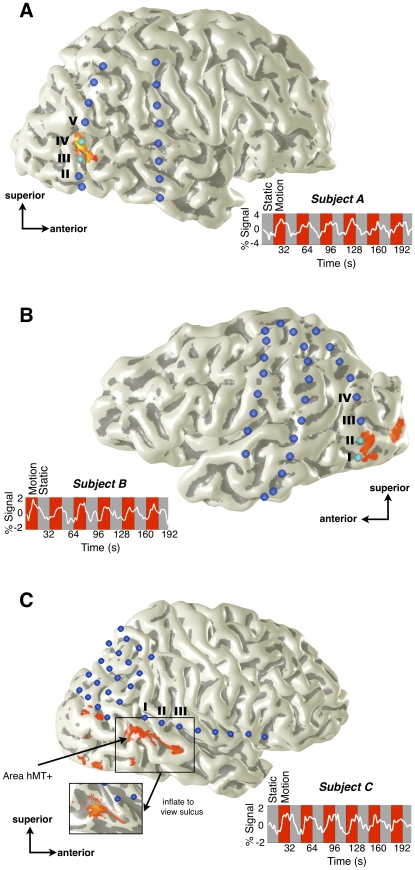
Overlap of intracranial electrodes with functional MRI localizer of area hMT^+^. Location of intracranial electrodes (blue disks) and the area of fMRI activation in the motion localizer task (orange-red) are shown for three subjects (A–C). Pairs of electrodes were electrically stimulated; cyan electrodes indicate those pairs between which electrical stimulation elicited reliable, lucid, illusory motion (see **Figure 3**). FMRI activation is thresholded at a *p*-value corresponding to a false discovery rate of 5% in each individual. FMRI time series, shown next to each subject’s 3D cortical surface, are extracted from the hMT^+^ region of interest and averaged across two runs.

In three subjects, functional imaging using fMRI independently revealed higher levels of blood-oxygenation-level-dependent (BOLD) responses bilaterally in the posterior inferior temporal sulcus when viewing moving, compared to static, visual stimuli (**Figure 1A–C**). This area of increased BOLD activation in response to moving visual stimuli was labeled area hMT^+^ in each individual separately (see Materials and Methods for details).

**Figure 2:**
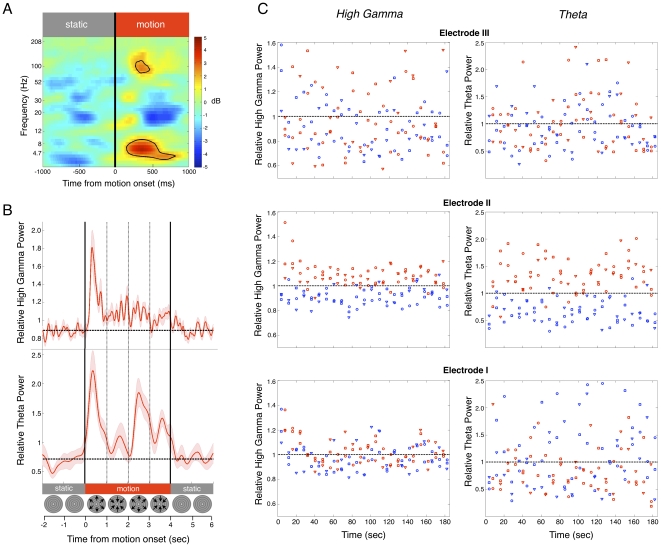
Electrophysiological response to the same motion stimuli as during fMRI. (A) Power spectrogram from electrode II in subject B during one representative run. Electrode II was the only electrode overlying the area of significantly increased BOLD activation during the motion stimulus (see Figure 1B). This electrode shows significantly increased power (denoted in decibels, dB) in the high-gamma and theta bands during motion compared to static (yellow-red). The significance threshold is FDR-corrected (q = 0.1, p<0.02). For presentation purposes, the spectrogram is smoothed over 3 frequency bands and 230 ms. (B) Temporal profiles of the relative power for high-gamma and theta bands in the same electrode during the same run. The power of high-gamma and theta band activity was normalized by the mean power (y = 1) within that band and across the run. The power in each band was scaled by these means. Vertical solid lines indicate transitions between static and motion, and vertical dotted lines indicated transitions between outward and inward motion of the stimulus. Horizontal dashed lines indicate the mean relative power during the static condition. The shading on the time courses indicates the standard error of the relative power. (C) The increase of high-gamma and theta power during the motion stimulus is consistent across individual trials and is selective to electrode II. Each red marker denotes the mean relative power across a single four-second trial of motion, while each blue marker denotes the mean relative power across a single four-second trial of static, for electrode II (middle row) and two neighboring electrodes (top and bottom rows, see Figure 1 for precise locations), across the duration of each run of the experiment. Different shapes (circles, squares, triangles) denote different runs of the experiment. Note that electrode I was near, but not overlapping with, the area of significantly increased BOLD activation to motion.

Intracranial electrophysiological recordings (ECoG) in subject B revealed a marked spatial overlap between the BOLD response and electrophysiological activity during the same task. During blocks of moving images, there was a significant increase in power specific to the theta (4–7 Hz) and high-gamma (50–120 Hz) bands (**Figure 2A**) only in the electrode directly overlapping with the fMRI-defined area hMT^+^. This electrophysiological signature is consistent with previous reports of the relationship between electrophysiological and BOLD measures [31]. Note that no ECoG recordings were performed in subject A.

The temporal profile of the power (**Figure 2B**) in the theta and high-gamma frequency bands shows several noteworthy findings. The profile of high-gamma and theta responses is very distinct after the first second of motion stimulus presentation. At the onset of the motion stimulus, the relative power of high-gamma band activity increases up to ∼190% of the power during the static stimulus and sustains an elevated power (∼120% of power during static stimulus) for the entire four seconds of the motion stimulus. The relative theta power is modulated at the frequency of the stimulus, with peaks in the theta power occurring approximately at the mid-point between transitions from inward to outward movement of the concentric circles. Interestingly, the peaks in theta power reach a higher level for outward than for inward motion, suggesting similarities in the response properties of our recorded theta modulation in the human brain to non-human primate neuronal tuning in MST, which shows a higher proportion of cells responsive to expansion than contraction [32].

Changes in the high-gamma and theta frequencies occur in individual four-second trials, only for the electrode overlapping with the area of significant BOLD modulation. For each electrode, we plotted the mean relative power for both the theta and high-gamma bands over each individual four-second trial (**Figure 2C**). These plots illustrate the clear separation of responses to motion and static stimuli in the mean relative power of the high-gamma and theta bands, only in electrode II (middle row, Figure 2C). For electrode II, 91% of motion trials show a response above the mean power of the high-gamma band (i.e. above y = 1 in **Figure 2C**), and 90% of static trials show a response below the mean relative power of the high-gamma band. Theta band responses during individual trials are similarly consistent (89% of motion trials above mean theta power and 94% of static trials below mean theta power). For both high-gamma and theta bands, the mean of the distribution during the motion condition is larger than the mean of the static condition (p<0.001, t-test; for all 3 experiments and each band).

Electrodes that did not overlap with areas of significant BOLD modulation failed to show significant electrophysiological activation in response to the same motion stimulus in ECoG recordings. In all other analyzed electrodes (N = 14), none of which overlapped fMRI-defined area hMT^+^, the distributions of responses to motion and static trials are not well-separated (p>0.15, t-test, for all 3 experiments and each band), with approximately equal numbers of points of each condition falling above and below the mean relative power (see Electrodes I and III in Figure 2C as examples). In subject C, intracranial electrodes were situated near the border of, but not within, fMRI-defined area hMT^+^ (Figure 1C). In this subject, we did not find any significant task-induced theta or high-gamma band activity in any intracranial electrodes, congruent with the idea that the electrophysiological and BOLD signals agree spatially.

### Electrical brain stimulation in hMT+ causes illusory visual motion

As part of routine brain mapping procedures conducted for clinical purposes, electrical stimulation was performed in all three patients. During this process, a weak and focal electrical current was delivered to the brain area located between two electrodes (i.e. bipolar stimulation) while subjects were lying comfortably in the hospital bed with their eyes open. Patients were generally unaware of the timing of electrical stimulus delivery, which also included interspersed sham stimulations. Subjects were asked to describe in detail all changes in perception or subjective experience during electrical stimulation. We define illusory visual motion percepts as any change in conscious visual perception that (a) involves the translocation of one or more parts of the visual environment across visual space and (b) is directly elicited by the electrical stimulation.

**Figure 3:**
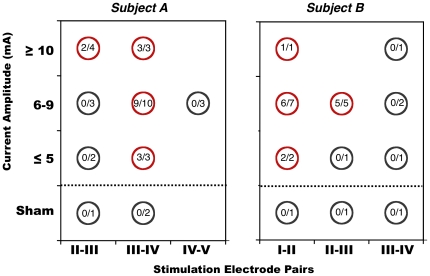
Electrical stimulation only over hMT^+^ evokes illusory motion. Red circles represent stimulation sites and amplitudes that elicited illusory motion at least once, while dark gray circles represent stimulation sites and amplitudes that did not elicit any illusory motion. Numbers inside circles represent the number of electrical stimulation trials evoking illusory motion over the total number of electrical stimulation trials with a particular pair of electrodes at that current amplitude. Sham indicates trials where no current was injected but the subject thought stimulation was taking place. Subject C did not perceive any illusory motion (not shown). The quality of the motion percept differed between subjects A and B but was highly consistent within each individual. No other stimulation sites elicited a percept of visual motion, even though all adjacent pairs of electrodes in the brain were electrically stimulated for clinical reasons. See **Figure 1** for electrode positions.

Reproducible, vivid, illusory visual motion percepts occurred when electrical charge was delivered through electrodes that were localized within the hub of fMRI activity corresponding to hMT^+^ in subjects A and B (**Figure 3**). The qualitative experience of the percepts was stereotyped within each individual regardless of stimulation intensity (1-12 mA) or duration (3–6 sec). The conscious illusory experiences in subjects A and B were similar but not identical. Electrical stimulation of right hMT^+^ in subject A caused displacement and transposition of the entire visual field to the left (i.e. optical allesthesia). This reported illusory percept of the visual field “jumping” to the left was spontaneously generated and present even with eyes fully deviated in left lateral gaze. In subject B, electrical stimulation of left hMT^+^ caused an illusory percept of objects moving in the contralateral (right) upper visual field as if they were “vibrating” (subject’s word). For example, while looking at the experimenter’s face, the subject reported that “the top right corner of the face is vibrating”. The effect was limited to the subject’s upper right visual field quadrant. Interestingly, when the subject’s eyes were closed and he was asked to imagine an object he had just seen, the imagined object was reported as “vibrating” during electrical stimulation and not during sham stimulation.

The intensity of the illusory experiences in both subjects was not subtle. The subjects volunteered their descriptions readily and seemed to be completely captivated by the intensity of the experience. Importantly, subjects successfully kept fixation during electrical stimulation trials. Exhaustive direct inspection of video recording obtained during electrical stimulation did not reveal any macroscopic eye movements in either subject during electrical stimulation (see Video S1; note the consideration of imperceptibly small eye movements in Discussion).

The total number of electrical stimulation trials at each site and the number of times a motion percept was elicited at that site is shown in **Figure 3**. Across all subjects, stimulation directly over hMT^+^ (III–IV in Subject A, I–II in Subject B, see Figure 1 for locations) elicited illusory motion percepts in 92% (24 of 26) of trials (**Figure 3**). Electrical stimulation at sites directly neighboring hMT^+^ (II–III and IV–V in Subject A, II–III in Subject B) elicited the illusory motion in 39% (7 of 18) of trials, but these positive trials only occurred at the highest stimulation amplitudes tested. At these neighboring locations, one of the two bipolar electrodes was overlapping hMT^+^. In contrast, stimulation at all other cortical locations, where neither stimulating electrode overlapped hMT^+^, elicited illusory motion 0% (0 of over 100 trials) of the time. Sham stimulation trials, which were interspersed between hMT^+^ stimulation trials and did not involve current delivery, also did not elicit any illusory motion (0 of 6 trials).

Illusory motion was not elicited by stimulation at any electrode sites in subject C, who had electrodes positioned adjacent to, but not within, fMRI-defined area hMT^+^ (**Figure 1C**). Together with the results from subjects A and B, these negative findings in subject C further suggest a high degree of spatial congruence between fMRI and electrophysiological responses to motion and conscious perceptions of motion elicited by electrical stimulation. Although not the focus of the current report, other perceptual illusions (such as an urge to move the contralateral hand, or tingling in the contralateral side of the body) occurred at some other electrode sites. None of these percepts were related to visual motion perception.

## Discussion

We report that electrical stimulation of functionally defined cortical area hMT^+^ causes reproducible illusions of visual motion. This illusory visual motion was only elicited when the site of electrical stimulation was precisely overlapping with the area of fMRI activation, defined independently in each subject in response to visual motion stimuli (**Figure 1**). Moreover, the electrophysiological activity recorded by ECoG during the same task was clearly limited to the electrode overlapping the area of fMRI activation (**Figure 2**).

We interpret these results in the context of previous human and non-human primate studies that have shown the causal necessity of area MT for motion perception. Our results show, for the first time, that altering neural activity in hMT^+^ by electrical charge delivery is sufficient for producing complex positive illusions of visual motion (**Figure 3**). They also provide converging evidence from three different methodologies (i.e. fMRI, ECoG, and EBS) that allows inferences regarding the electrophysiological basis of the fMRI signal and the relevance of fMRI and ECoG correlates of a perceptual task to human conscious perception.

### Necessity and sufficiency- a causal link between the activity of the hMT+ network and subjective visual motion perception

A substantial body of previous research has provided strong evidence to support correlations between hMT^+^ activity and visual motion perception [7]. Causal links between MT activity and motion direction discrimination judgments have been demonstrated in the non-human primate [5], but studies in non-human primates have limited ability to address the subjective perceptual experience produced by experimental alterations of MT neuronal activity. The loss of cortical tissue surrounding the anatomical location of MT/hMT^+^ has been shown to produce loss of motion sensitivity in non-human primates [9] and akinetopsia (motion blindness) in humans [11], both negative symptoms. Similarly, transcranial magnetic stimulation (TMS) to hMT^+^ can lead to transient loss of motion sensitivity [13], [15]. These prior studies provide evidence for the necessity of MT/hMT^+^ in motion perception.

While disruption of function (negative effect supporting necessity) can occur following lesions or TMS, positive percepts (supporting sufficiency) can only be achieved by altering, rather than stopping, the activity of a critical network. Reports of positive percepts of motion are much more rare and have not been linked to human area MT^+^ as defined by BOLD fMRI. The placement of intracranial electrodes in the human brain is a unique opportunity to observe the effects on conscious perceptual experience during alterations of neural activity. Reproducible and consistently elicited conscious motion percepts caused by electrical charge delivery to hMT^+^, as reported here, satisfy conditions of sufficiency. That is, altering the neural activity within hMT^+^, and the network it is connected with, is sufficient for producing vivid subjective motion percepts.

In one previous study, electrical stimulation at the border of hMT^+^ [29] failed to elicit any percept (similar to our finding in subject C). Blanke et al [17] also failed to produce positive illusory visual percepts during electrical stimulation of the temporo-parietal region in a single patient, but it is noted that the posterior extension of their electrode grid only covered the anterior portions of the junction between the inferior temporal sulcus (ITS) and the ascending limb of the ITS, where area hMT^+^ is generally thought to be located. Also, no fMRI or ECoG measures of visual motion perception were obtained. In addition to exact location, precise electrical stimulation parameters may be crucial in determining whether positive or negative perceptual phenomena occur. The lack of positive perceptual phenomena in these previous studies are in line with our own null result in subject C, and can be explained by our observations in subjects A and B that the positive phenomenon of illusory visual motion is elicited only if the site of EBS is co-localized precisely with the brain site that shows positive functional response (identified by fMRI or ECoG) during visual motion perception. This need for functional localization is clear when considering the individual variability of the location of hMT^+^ with respect to anatomical landmarks [33].

### Visual imagery is affected by electrical stimulation of hMT+

In our experiment, we asked subject B to close his eyes and imagine a recently viewed object “in his mind’s eye” while electrical charge was delivered to hMT^+^. Interestingly, the subject reported the same visual motion illusion (“vibrating”, or oscillatory left-right motion of the imagined object) caused by electrical stimulation. In contrast, sham stimulation during imagery trials elicited no positive reports by the subject (i.e. he did not see any change in the mental image; see Video S1). Therefore, the percept produced by electrical stimulation of hMT^+^ affects a mental image similarly to a real visual image. This finding lends support to the hypothesis that mental imagery may be an emulation of perception and that the neurons that code for a mental image may be the same as, or overlap with, those used in visual perception [34], [35].

### Propagating electrical charge within selective anatomical networks

The spatial spread of electrical charge is an important consideration for interpreting results from EBS experiments. Although little is known about the effect of electrical stimulation of the cerebral cortex in the human brain, the emerging evidence from cortical micro-stimulation (micro-EBS) [36] and deep brain stimulation (DBS) [37] in mammalian brains strongly suggests that the electrical charge delivery is more likely to recruit neural fibers whereas the activity of neurons in the stimulated area is either unchanged [37], blocked through depolarization blockade [38], or only altered in a sparse and distributed set of neurons [36], [39]. Reliable recruitment of neural fibers will lead to propagation of electrical activity along the afferent or efferent fibers and will reach the brain regions that are connected with the stimulated area of the brain [40]. Given that each region of the brain has selective neuroanatomical connectivity with cortical and subcortical structures, the propagation of electrical activity will only affect the activity of a selective neuroanatomical network. Thus it may be difficult to compare the functional effect of EBS, as used in brain mapping procedure, to the effect of TMS, micro-stimulation, DBS, or structural lesioning. In other words, during brain mapping, a volley of 50 Hz signals may cause depolarization blockade (i.e. impairment of function) in the actual target of electrical stimulation but, at the same time, the volley of 50 Hz electrical signals recruits a selective neuroanatomical network in the gamma band frequency. In our experiments, it is possible that hMT^+^ may have been blocked by the depolarization blockade, but in conjunction with the recruitment of its selective neuroanatomical network (such as V1), the manipulation seems to be sufficient to lead to a subjective experience of visual motion. Given that the network is recruited artificially with 50 Hz signals, the resulting subjective experience is an illusion of visual motion when there is no real motion in the visual field (i.e. a positive phenomenon). It is interesting to note that back-propagation of signals from hMT^+^ to V1 is thought to be necessary for visual awareness of motion percepts [41].

Whether the effect of EBS is excitatory or inhibitory depends on stimulation frequency, and stimulation frequency at 50 Hz, as in our study, is more likely to be inhibitory [42]. Inhibitory effects on connected brain areas may be as relevant as the excitatory effect of electrical stimulation for causing positive illusory phenomena. It is likely that the inhibitory effect of EBS on the areas connected to MT, such as visual areas V1 to hV4 and parts of parietal cortex [43], which are involved in maintaining the stability of the visual world [44]–[47], may result in instability of visual images and hence the illusion of motion.

Because the EBS in our study was performed in a purely clinical setting for clinical diagnosis, which does not easily accommodate research stimuli/procedures, we were unable to test the ability of subjects to perceive normal visual motion during electrical charge delivery to area hMT^+^. However, given the magnitude of the illusory percept caused by the EBS, it is more than likely that the subjects would have failed to perceive normal visual motion during the procedure. Therefore, our finding of positive illusory percept is not in conflict with the previous findings of impairment in visual motion perception during electrical stimulation of hMT^+^.

### Mechanistic interpretations of different perceptual experiences

The precise perceptual experiences reported by the two subjects differed and would be difficult to predict *a priori*. Nevertheless, previous literature suggests that both types of percepts are supported by hMT^+^ activity. Subject A’s percept is qualitatively similar to the phenomenon of “apparent motion”. This phenomenon describes the perception of jumping motion between two sequentially blinking stationary stimuli separated in space. In humans, hMT^+^ activity, and perhaps feedback from hMT^+^ to early visual cortex, correlates with the perception of apparent motion [48], [49]. As for subject B’s percept, there is also evidence that MT in monkeys and humans is required for perceiving lateral oscillatory motion [50].

We further propose that the percepts in subjects A and B may both be related to the role of hMT^+^ and its selective neuroanatomical network in supporting the stability of the visual world during normal vision. Specifically, the reported illusion in subject A is reminiscent of descriptions of a shifting visual world after retrobulbar paralysis of the eye muscles [51]. The similarity of these descriptions, along with the proposed roles of MT and parietal regions during saccadic eye movements [44], [46], suggests that electrical alteration of activity in hMT^+^ in subject A may have caused alteration of activity in its anatomical network (i.e. synthetic and erroneous signal from hMT^+^ to its connected parietal areas). These synthetic signals could be interpreted by the receiving areas as a corollary discharge for an eye movement that did not, in fact, take place. A corollary discharge would be expected to result in a shifting visual world in preparation for an eye movement [44], [47].

The experience of visual jitter in subject B may be related to MT’s normal active role in suppressing movement of the visual world due to microsaccadic eye movements [45], [52]. Introducing spurious signals through electrical stimulation of the set of neurons underlying these computations would conceivably alter the relationship between MT signals and ongoing microsaccadic eye movements, leading to perceptions of microsaccades in a restricted region of the visual field. (The effect would be spatially localized because hMT^+^ is organized retinotopically—see [53]). Currently, we cannot distinguish between such an indirect effect and the possibility that the alternating electrical current from EBS is directly interpreted as alternating left-right motion in this subject. However, we can exclude the possibility that EBS directly caused eye movements that explain the percept because the percept was limited to one quadrant of the visual field, while an induced, microscopic nystagmus would be equally salient in all parts of the visual field.

We note that all subjects were able to keep visual fixation during electrical stimulation trials (Video S1), although we cannot exclude the possibility that electrical stimulation caused imperceptible eye movements. However, such small eye movements would be unlikely to explain the large visual motion percepts experienced by subject A, or the spatially localized percepts (within a visual field quadrant) experienced by subject B. Even if small eye movements were to explain the reported percepts, it is interesting that they would have occurred only with electrical stimulation of hMT^+^.

The differences in the percepts reported by the two subjects might be attributed to the involvement of different sub-regions of hMT^+^, MT and MST, each of which could have their own network connectivity. While our current methods did not allow us to specifically address whether different sub-regions were stimulated in each subject, future studies can incorporate stimuli intended to differentiate between MT and MST [3] to test this hypothesis. Finally, since electrode grids were implanted in the right hemisphere of subject A and the left hemisphere of subject B, the differences in reported perceptions may also be due to a left-right hemispheric functional asymmetry in the affected networks.

### Linking fMRI, ECoG, and EBS

ECoG recordings are a field potential aggregated from approximately 5x10^5^ neurons underlying each electrode [54], similar to the number of neurons in an fMRI voxel (10^5^ neurons/mm^3^×∼5–30 mm^3^ voxel size). This similar spatial resolution to fMRI, in conjunction with the similarity in the signal type to the local field potential (LFP), puts ECoG recordings in a unique position to link fMRI BOLD findings in humans to LFP responses in non-human primates [30]. The ECoG response to the same motion stimulus as used for fMRI was limited to the theta and high-gamma bands, suggesting that these particular frequency bands correlate with the hMT^+^ BOLD signal response. Future studies can test the generality of these findings in more subjects.

We note the strong similarity of our ECoG recordings from hMT^+^ (**Figure 2A**) to LFP recordings from area MT in the non-human primate using microwire electrodes (Figure 3 in [55]). In both cases, there is increased power in the high-gamma band (∼50–120 Hz) at the onset of the stimulus. In our recordings, using a long four-second stimulus, the strong high-gamma band response decreases somewhat after approximately 500 ms. The theta power is sustained at a high level throughout the stimulus (**Figure 2B**), although it is also temporally modulated by the stimulus. Such differential dynamics of signals across frequency ranges will be an interesting point of study in the future. Combining the multiple methodologies of fMRI, ECoG, and EBS provides an especially powerful set of interrelated findings to help understand specific functions of cortical areas.

### Epileptic brains

Although our results were obtained in patients with epilepsy, we believe the results are unlikely to be explained by pathological factors. As noted, area hMT^+^ was void of any epileptiform activity in all three patients, and data from any electrodes showing epileptic activity were excluded in our electrophysiological analysis. The positive illusory percepts were also recorded without the presence of any after-discharges. Our study included only three subjects, but it should be noted that the posterior regions of the brain are rarely implanted with electrodes and thus intracranial recordings from hMT^+^ are uncommon. Restrictions due to the clinical setting of this research provided other challenges as well. We were not able to perform ECoG recording from the hMT^+^ electrodes in Subject A because the EBS procedure was performed shortly before surgery and we could not delay the surgery in order to obtain those recordings.

### Conclusions

Taken together, our findings are consistent with studies in non-human primates suggesting a crucial role of area MT and its interconnected network in conscious motion perception. We demonstrate that electrical stimulation of area hMT^+^, as defined by fMRI and verified by electrophysiological responses in individual subjects, elicits a conscious experience of visual motion in awake human subjects. In the context of previous research, our results show that the hMT^+^ network circuitry is both necessary and sufficient for producing conscious motion percepts. The spatial agreement of fMRI and electrophysiological measures allows inferences about the link between these stimulus-evoked signals and their ultimate relation to conscious visual perception when the activity of the same part of the brain is electrically modulated.

## Materials and Methods

### Ethics Statement

Our study was approved by the Stanford University IRB Office for Protection of Human Research Subjects. All subjects signed informed consent for participation in our research study.

### Subjects

Our subjects were three patients (1 male, 2 female) undergoing epilepsy surgery for intractable epilepsy. Our study did not cause additional risk to any participants, and the intracranial procedures were conducted entirely for clinical reasons to localize the source of epileptic discharges. Our diagnostic studies revealed no pathological activity in hMT^+^. Patient A was diagnosed with multifocal epilepsy originating from frontal and posteromedial regions, whereas patients B and C were diagnosed with epileptic foci in the medial (but not lateral) parieto-occipital region, after resection of which, both subjects, to date, remain seizure free.

### Functional Magnetic Resonance Imaging (fMRI)

Localizer sessions were aimed at identifying motion-responsive areas. The stimulus consisted of a set of concentric dark gray circles on a gray background. The stimulus alternated between static and moving in blocks of 16 sec. During motion blocks, the circles expanded and contracted at a rate of 0.5 Hz (i.e. one full expansion and contraction every two seconds). Each run (n = 2) lasted 208 secs (192 secs in subject B) and included 6 blocks of motion and 7 blocks of static stimuli (6 static in subject B). Subjects fixated on a white dot in the center of the screen and pressed a button anytime the fixation dot randomly flashed red. All subjects performed this independent task at near 100% accuracy, indicating stable fixation. Functional magnetic resonance images were acquired on a 3T GE MRI scanner and an 8-channel volume head coil using a spiral-trajectory pulse sequence [56] with the following parameters: one shot, TR = 2000 ms, TE = 30 ms, flip angle = 77°, FOV = 220 mm, voxel size = 1.72×1.72×2 mm^3^ in subjects A and C, 3×3×2.5 mm^3^ in subject B. Twenty-one oblique slices covering occipital and temporo-parieto-occipital cortex were prescribed approximately along the AC-PC plane.

We analyzed fMRI data using the freely available, open-source mrVista software package (http://vistalab.stanford.edu/software/). The acquired BOLD signal from each voxel was first divided by its mean in order to compute a time series of percent modulation. High-pass temporal filtering was used to deduct baseline drifts from the time series. Small motion artifacts within and across scans were corrected using an affine transformation of each temporal volume in a data session to the first volume of the first scan [57]. The data were analyzed on a voxel-by-voxel basis using a general linear model (GLM) that modeled the BOLD signal using a two regressors (motion and static), with an additional DC regressor for each run to account for shifts in baseline. Statistical maps were computed as voxel-wise t-tests between the motion and static conditions. Area hMT^+^ was defined by the contrast motion > static at a statistical threshold equivalent to a false discovery rate of 5% (q = 0.05) in each individual subject. The resulting statistical contrast maps were interpolated to the T1-weighted volume anatomy and restricted to gray matter layers. These maps are projected onto a cortical surface mesh (consisting of the surface along the gray-white boundary) for visualization. In subject A, the fMRI hMT^+^ localizer was performed post-surgically, while subjects B and C participated in the same localizer session before electrode implantation.

### Electrode Localization

We used MS08R-IP10X-000 strips and IG64C-SP10X-0TB grids made by AdTech Medical Instrument Corporation (http://www.adtechmedical.com) for recording and stimulation in our subjects. These electrodes have the following parameters: 4 mm flat diameter contacts with 2.3 mm diameter of exposed recording area (4.15 mm^2^) and inter-electrode distance of 1 cm. Post-surgical computed tomography (CT) images indicating the location of electrodes were aligned to preoperative T1-weighted structural MRI images using a mutual-information algorithm, implemented in SPM5 (http://www.fil.ion.ucl.ac.uk/spm). The electrodes were easily identified in the CT scans and their locations were manually marked. These images were visualized using ITKGray, a segmentation tool based on ITKSnap [58]. The resulting images were manually aligned to 3D mesh renderings of the T1 anatomical images produced using mrVista, on which the fMRI activation is displayed, thereby conserving the electrode to T1 anatomical image alignment. This procedure allowed us to construct 3D visualization of electrode locations relative to each patient’s cortical anatomy within a few millimeters (<∼3 mm) in error. The accuracy of estimated electrode sites was also validated by digital photos, obtained intraoperatively.

### Electrophysiological Recording and Analysis

After implantation of the electrodes and post-surgical stabilization, the hMT^+^-localizer task was administered to the patients for ECoG recordings (patients B and C only). This task was identical to the one described for fMRI, except that blocks were 4 seconds in length instead of 16 because of the increased temporal resolution of ECoG over fMRI. There were 22 blocks of motion and 23 blocks of static, giving a run time of 180 s. Subject B completed three runs, and subject C completed two runs. We recorded signals at 3051.8 Hz through a 128-channel recording system made by Tucker Davies Technologies (http://www.tdt.com/). Off-line, we applied a notch filter at 60 Hz and harmonics to remove power line noise. We removed channels with epileptic activity, as determined by the patient’s neurologist. To visualize electrophysiological responses, we created event-related spectral perturbation (ERSP) maps based on the normalized power of electrophysiological activity during each condition. A Hilbert transform was applied to each of the 42 bandpass filtered time series to obtain instantaneous amplitude and power [59]. Using the Hilbert-transformed time series, time-frequency analysis was performed for event-related data. We logged the onset and duration of each trial via photodiode event markers for each experimental condition time locked with the ECoG recording. Event markers were used to align and average power at each frequency band over repeated trials for each condition to create ERSP maps. The ERSP was scaled by the total mean power at each frequency in order to compensate for the skewed distribution of power values over frequencies and the result was converted to decibel units.

In order to test the significance of changes in ERSP, we compared each ERSP frequency-time point with a constructed “null” ERSP. We first generated a surrogate data set by transforming the original instantaneous power time series into the Fourier domain and adding random phases, resulting in a surrogate of instantaneous power that has randomized phase but preserved amplitude. Therefore, the first and second order moments of the surrogate remained unchanged but its local temporal structure was removed [60]. A “null” ERSP was then constructed from the surrogate data with the same number of trials (randomly selected) as the condition of interest. We constructed a set of “null” ERSPs by iterating the surrogate procedure 470 times (e.g. for the presented ERSP in **Figure 2A**, we generated 47 surrogate data sets and for each set, we shuffled the surrogate events 10 times). We expect that the distribution of the “null” ERSP at each frequency-time point approaches a Gaussian distribution with sufficient iterations (law of large numbers). We tested the Gaussianity of the constructed distribution by monitoring kurtosis. We kept the absolute value of the distribution kurtosis below 0.5 (the kurtosis of Gaussian is zero) by increasing the number of iterations of the surrogate procedure. Following this procedure, we used a normal distribution to fit the “null” ERSP at a given frequency for one cycle period in order to estimate its mean and standard deviation. We shifted and scaled the ERSP at each frequency-time point relative to the obtained mean and standard deviation (Z-score). We then converted the normalized ERSP (Z-scores) to p-values. Finally, we used a false discovery rate method to correct for multiple comparisons and to set a significance threshold level for each subject, electrode and condition. Tests of significance for increases or decreases in the ERSP map were performed separately. The parameters for presented ERSPs are: (q = 0.1; p-values for the increase and decrease are 0.02 and 0.001, respectively).

### Electrical Brain Stimulation (EBS)

Electrical stimulation was performed as part of routine clinical procedure of brain mapping to determine areas of hyperexcitability whose stimulation causes the patient’s typical behavioral seizures, and to determine the function of each brain region before making a decision about the extent of epilepsy surgery [25]. Electrical charges used (50 µC/cm^2^/pulse) in each patient were within the safety parameters and appreciably less than the ones used in older classical studies by Penfield and colleagues (∼700 µC/cm^2^/pulse). Stimulation was performed using the following parameters: Square wave currents from 1 to 12 mA at 50 Hz and with a pulse width of 200 µs. The impedance of these electrodes is measured to be approximately 400 Ω at 1 kHz [61]. Subjects were comfortably lying in their hospital bed during bipolar electrical stimulation, with their eyes open (except where noted) and fixated on an object in the room. Eye movements were monitored by video recordings. Care was taken not to influence subjects’ reports of perceptions by asking open-ended questions (“Did you hear, see, or feel anything strange?”) and by including the same questions during sham stimulation trials.

## Supporting Information

Video S1 This supplementary video file shows how stimulation of the hMT+ in two patients with implanted intracranial electrodes causes illusion of visual motion.
(MP4)
